# Effects of Transendocardial Stem Cell Injection on Ventricular Proarrhythmia in Patients with Ischemic Cardiomyopathy: Results from the POSEIDON and TAC‐HFT Trials

**DOI:** 10.1002/sctm.16-0328

**Published:** 2017-03-02

**Authors:** Archana Ramireddy, Chad R. Brodt, Adam M. Mendizabal, Darcy L. DiFede, Chris Healy, Vishal Goyal, Yahya Alansari, James O. Coffey, Juan F. Viles‐Gonzalez, Alan W. Heldman, Jeffrey J. Goldberger, Robert J. Myerburg, Joshua M. Hare, Raul D. Mitrani

**Affiliations:** ^1^ Cardiovascular Division, Department of Internal Medicine, University of Miami Miller School of MedicineMiami, FLUSA; ^2^ Interdisciplinary Stem Cell Institute, University of Miami Miller School of MedicineMiamiFloridaUSA; ^3^ EMMES CorporationRocklandMDUSA

**Keywords:** Stem cells, Cardiomyopathy, Heart failure, Cardiac arrhythmias, Ventricular tachycardia

## Abstract

Transendocardial stem cell injection in patients with ischemic cardiomyopathy (ICM) improves left ventricular function and structure but has ill‐defined effects on ventricular arrhythmias. We hypothesized that mesenchymal stem cell (MSC) implantation is not proarrhythmic. Post hoc analyses were performed on ambulatory ECGs collected from the POSEIDON and TAC‐HFT trials. Eighty‐eight subjects (mean age 61 ± 10 years) with ICM (mean EF 32.2% ± 9.8%) received treatment with MSC (*n* = 48), Placebo (*n* = 21), or bone marrow mononuclear cells (BMC) (*n* = 19). Heart rate variability (HRV) and ventricular ectopy (VE) were evaluated over 12 months. VE did not change in any group following MSC implantation. However, in patients with ≥ 1 VE run (defined as ≥ 3 consecutive premature ventricular complexes in 24 hours) at baseline, there was a decrease in VE runs at 12 months in the MSC group (*p* = .01), but not in the placebo group (*p* = .07; intergroup comparison: *p* = .18). In a subset of the MSC group, HRV measures of standard deviation of normal intervals was 75 ± 30 msec at baseline and increased to 87 ± 32 msec (*p* =.02) at 12 months, and root mean square of intervals between successive complexes was 36 ± 30 msec and increased to 58.2 ± 50 msec (*p* = .01) at 12 months. In patients receiving MSCs, there was no evidence for ventricular proarrhythmia, manifested by sustained or nonsustained ventricular ectopy or worsened HRV. Signals of improvement in ventricular arrhythmias and HRV in the MSC group suggest a need for further studies of the antiarrhythmic potential of MSCs. Stem Cells Translational Medicine *2017;6:1366–1372*

**Significance Statement**

Our study is a novel post hoc analysis which examines potential pro‐ and anti‐arrhythmic effects from transendocardial injection of mesenchymal stem cells (MSCs) in patients with ischemic cardiomyopathy. By combining patient populations from two prospective, randomized controlled trials (POSEIDON and TAC‐HFT) and examining ventricular ectopy and heart rate variability, we were able to study a group of patients who received MSCs and compared them to groups who received placebo and bone marrow‐derived mononuclear cells. The safety and efficacy of stem cell implantation has been explored in prior studies, but the potential arrhythmogenicity of this MSC therapy and how it compares to placebo injections have not previously been studied on this scale. Our study did not show any pro‐arrhythmia with MSC implantation, though there were important trends demonstrated between improvement of arrhythmias and this stem cell population in comparison to placebo or bone marrow derived mononuclear cells. These findings are important in that they beg the need for further large prospective studies to evaluate the anti‐arrhythmic potential of mesenchymal and other newer cell‐based therapies.

## Introduction

Heart failure remains an epidemic with prevalence rates estimated between 0.4 and 2% in Europe and the U.S. 1. Based on recent projections, the prevalence of heart failure is expected to increase from approximately 6 million to > 8 million Americans by 2030 2. Transendocardial stem cell injection (TESI) is a novel regenerative therapy with promising results for these patients 3, 4, 5, 6, 7.

Randomized controlled studies have demonstrated improvement in structural and hemodynamic parameters using different delivery strategies for a variety of cell types 4, 5, 6, 7, 8. Patients with ischemic cardiomyopathy (ICM) are at risk for sudden cardiac death 9. Although an increasing proportion of cardiac arrests are being attributed to asystole and pulseless electrical activity, ventricular tachycardia (VT), and ventricular fibrillation (VF) lead to cardiac arrest in a substantial number of cases 10, 11, 12. Structural risk factors for VT/VF include myocardial scar size, left ventricular ejection fraction, and chamber volume 13. Other prognostic risk factors for VT/VF and sudden cardiac death include but are not limited to frequent ventricular ectopy, nonsustained VT, prolongation of QT interval, increase in QT dispersion, and abnormal heart rate variability 9, 11, 14. It is unknown whether TESI of bone marrow‐derived mesenchymal stem cells (MSCs) mitigate, exacerbate, or do not affect ventricular arrhythmias. If stem cells establish areas of heterogeneous conduction and repolarization upon injection, the therapy might potentially be proarrhythmic 15, 16, 17. Prior studies in humans have shown lack of proarrhythmia 18, 19; however, these studies used bone marrow cells.

Our hypotheses were that MSC implantation was not associated with an increase in ventricular ectopy (VE) nor worsening of an important marker of ventricular arrhythmias such as heart rate variability (HRV). A secondary objective was to determine whether there were any signals of antiarrhythmic effect from MSC implant in the heart.

## Materials and Methods

POSEIDON (A Phase I/II, Randomized Pilot Study of the Comparative Safety and Efficacy of Transendocardial Injection of Autologous Mesenchymal Stem Cells Versus Allogeneic Mesenchymal Stem Cells in Patients With Chronic Ischemic Left Ventricular Dysfunction Secondary to Myocardial Infarction) and TAC‐HFT (Transendocardial Autologous Mesenchymal Stem Cells and Mononuclear Bone Marrow Cells in Ischemic Heart Failure Trial) were Phase I/II transendocardial MSC trials performed in patients with ICM 5, 6. Post hoc analyses of the combined datasets and ambulatory ECG monitors from these trials were performed. In the POSEIDON study, 31 patients were enrolled at the University of Miami, Miller School of Medicine (Miami, Florida) and the Johns Hopkins University School of Medicine (Baltimore, Maryland) between April 2, 2010 and September 14, 2011. In TAC‐HFT, 59 patients were randomized at the University of Miami from September 1, 2009 to July 12, 2013. All patients provided written informed consent for the Institutional Review Board‐approved protocols. The National Heart, Lung, and Blood Institute Gene and Cell Therapy Data and Safety Monitoring Board, local Institutional Review Boards, and the POSEIDON and TAC‐HFT research groups were each responsible for the safety and protection of the participating patients.

The enrollment criteria for either trial included LV dysfunction (ejection fraction < 50%) and history of ICM. Exclusion criteria included limited life expectancy (< 1 year), glomerular filtration rate of less than 50 ml/min/1.73 m^2^, serious radiographic contrast allergy, clinical requirement for coronary revascularization, history of a life‐threatening arrhythmia in the absence of an implanted defibrillator, or recent sustained ventricular arrhythmias requiring defibrillator therapy. Patients with persistent atrial fibrillation were also excluded.

In POSEIDON, patients were randomized to injections of various doses (20, 100, 200 million cells) of allogeneic or autologous bone marrow‐derived MSCs. In the TAC‐HFT study, patients were randomly assigned into 3 groups: autologous bone marrow mononuclear cells (BMCs), bone marrow‐derived autologous MSCs (200 million cells), or placebo. The stem cells or placebo were delivered by transendocardial injection to 10 sites in the border zone of infarcted myocardial territory as previously described 5, 6, 20. In this post hoc analysis, 29 patients from the POSEIDON trial were combined with the autologous MSC group of TAC‐HFT (19 patients) to create 3 groups: MSC (*n* = 48), Placebo (*n* = 21), and BMC (*n* = 19). Patients in both trials had been optimized in terms of evidence‐based medical and device therapy. One of the patients from the POSEIDON trial was excluded from this analysis because the patient had an intraventricular thrombus and thus never received any stem cell therapy. Another patient from POSEIDON was excluded as the raw data from the Johns Hopkins University were not available.

Patients underwent 24 to 48 hours ambulatory ECG monitoring at baseline after achieving optimal medical therapy, 4 continuous days after injection, 2 weeks post‐injection, monthly up to 6 months, and at 12 months of follow‐up. The ECG recordings were downloaded and analyzed using the Impresario Holter Analysis System by Del Mar Reynolds Medical, Inc. (Copyright 2001). Two cardiologists from the University of Miami Health System verified all tracings by visual inspection for accuracy of software interpretation of paced beats, ectopic beats, and artifact. A separate third cardiologist (R.D.M.) adjudicated any differences in rhythm interpretation between the first two readers.

HRV analysis was performed solely on patients from the POSEIDON trial, as raw data from the Impresario Holter System were available only in these patients. Of the 31 patients who were originally enrolled in the POSEIDON trial, 1 patient was excluded due to a left ventricular thrombus, and 6 were excluded due to unavailability of raw data. Predominant atrial pacing in 2 subjects and excessive noise/artifact in 1 subject precluded accurate HRV analysis. Therefore, 21 patients with complete and authenticated monitoring data were included in the HRV analysis. The ambulatory ECGs were analyzed utilizing the Impresario software for HRV parameters, focusing on the following time domain methods 21: (a) SDNN—the standard deviation of all normal sinus R‐R intervals, and (b) RMSSD—the root mean square of the difference between coupling intervals of adjacent R‐R intervals.

The indices of HRV were compared with functional and anatomic markers, such as ejection fraction, end‐diastolic volume, end‐systolic volume, and sphericity index 5. Contrast‐enhanced CT scanning was used to assess global LV function and volumes. An arbitrary cutoff of significant change was assigned as any increase in ejection fraction, 5% decrease in end‐diastolic volume, 5% decrease in end‐systolic volume, and 10% decrease in sphericity index. Improvement of HRV between groups with functional and anatomic improvement compared to those without improvement was then analyzed.

Eighty‐eight patients with usable ambulatory ECG monitors were included in VE analysis. We defined VE as the number of premature ventricular complexes (PVCs) recorded by an automated counter and normalized to a 24‐hour period. A VE run was defined as three or more consecutive PVCs. A subgroup analysis of patients with greater than or equal to 1 VE run in a 24‐hour period was also performed. Both POSEIDON and TAC‐HFT showed a significant decrease in scar size at 12‐month follow‐up in the MSC group, respectively, which may be related to change in VE 5, 6. Thus, the percent change in scar size was analyzed for the Placebo, BMC, and combined MSC groups. Patients without baseline or 12‐month data for scar size or ventricular ectopy were excluded from this analysis, yielding a total of 69 patients for comparison—15 patients who received Placebo, 13 patients who received BMCs, and 41 patients who received MSCs from both the POSEIDON and TAC‐HFT studies. The percent change in scar size was compared to the percent change in ventricular ectopy from baseline to 12 months for all patients, and linear regression analysis was used for each group.

The autologous and allogeneic MSC groups were combined for HRV analysis. The data for HRV were represented using means and standard deviations. Determination of statistical significance was performed using Student's *t* tests and repeated measures ANOVA. *p* values less than .05 were considered significant. Because of the large variability within the ventricular ectopy data over several orders of magnitude between patients, logarithmic transformation was utilized in an effort to normalize the data. However, as the data remained skewed post‐transformation, medians and IQR were used to summarize the nonparametric distributions. ANOVA and χ^2^ tests were used to determine differences in baseline characteristics between the 3 groups (MSC, BMC, placebo). Wilcoxon Signed Rank tests were used to determine statistical significance of change in ventricular ectopy from baseline to 12‐month follow up, and Kruskal–Wallis tests were used for intergroup comparisons.

## Results

Eighty‐eight patients (mean age 60.9 ± 9.9 years) with ICM (mean ejection fraction (EF) 32.2% ± 9.8%) from the POSEIDON and TAC‐HFT trials were included in our post hoc analysis. There were no differences in baseline gender, age, ejection fraction, mean heart rate, or use of antiarrhythmic drugs (Table 1). However, at baseline the BMC group had lower median VE per day at baseline (median 2.2, IQR [1.5–2.6]; *p* = .01), and the MSC group had higher median VE runs at baseline (median 0.5, IQR [0.0–1.5]; *p* = .02). No patients had ICD shocks and/or clinical VT events within 30 days prior to the implant procedure.

**Table 1:** Baseline demographics

	Placebo	BMC	Total MSC	*p* value
Total patients	21	19	48	
Gender				
Male (%)	20 (95.2%)	17 (89.5%)	44 (91.7%)	.72
Female (%)	1 (4.8%)	2 (10.5%)	4 (8.3%)	
Age (years)	60.7 ± 10.4	61.2 ± 8.4	59.9 ± 10.5	.99
Ejection fraction (%)	31.7 ± 9.5	35.9 ± 8.2	32.3 ± 10.5	.19
Sphericity index	0.48 ± 0.1	0.45 ± 0.1	0.49 ± 0.1	.39
Heart rate	71.3 ± 7.9	72.2 ± 8.9	73.8 ± 9.2	.55
NYHA class				
I	4 (22.2%)	5 (26.3%)	9 (18.8%)	.97
II	10 (55.6%)	10 (52.6%)	29 (60.4%)	
III	4 (22.2%)	4 (21.1%)	10 (20.8%)	
Device type (%)				
AICD	8 (38.2%)	10 (52.6%)	29 (60.4%)	.07
CRT‐ICD	3 (14.3%)	1 (5.3%)	10 (20.8%)	
None	10 (47.6%)	8 (42.1%)	9 (18.8%)	
Medications				
Beta blockers	20 (95.2%)	17 (89.5%)	44 (91.7%)	.79
ACEi/ARBs	16 (76.2%)	17 (89.5%)	42 (87.5%)	.40
Use of Anti‐arrhythmic medications	5 (23.8%)	8 (42.1%)	17 (35.4%)	.46

Abbreviations: ACEi/ARBs, ACE inhibitors/ angiotensin II receptor blockers; AICD, automatic implantable cardioverter‐defibrillator**;** BMC, bone marrow mononuclear cells; CRT‐ICD, cardiac resynchronization therapy‐implantable cardioverter‐defibrillator; MSC, mesenchymal stem cell NYHA, New York Heart Association.

There were two deaths, both of them attributable to sudden death. One patient had received MSCs, also had severe COPD and recent pneumonia and had presented with acute respiratory distress prior to PEA arrest. The other patient had been in the placebo group and died suddenly at home. There were appropriate ICD shocks for ventricular tachyarrhythmias in 4 patients and an inappropriate ICD shock in 1 patient for atrial fibrillation: 3 (16%) of the patients in the BMC group were shocked compared with 1 patient (2.1%) in the MSC group, and 1 (4.8%) patient in the placebo group (*p* = .08 comparing ICD shocks between BMC and MSC group; NS between all other groups). During the actual implant procedure, an additional 1 patient had an episode of VT requiring cardioversion which was attributed to nonspecific response to catheter manipulation within the left ventricle.

**Figure 1:**
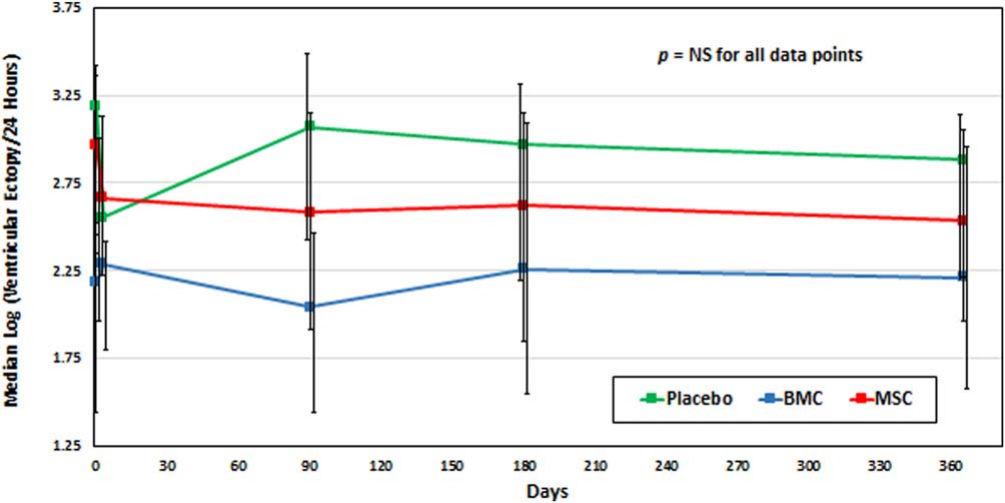
Change in log (ventricular ectopy/24 hours) from baseline to 12 months. Despite logarithmic transformation of the data, data remained skewed so nonparametric statistical analysis was used. There was no significant change in the acute phase after TESI or at 12 months post‐TESI in any of the three groups. Abbreviations: BMC, bone marrow mononuclear cells; MSC, mesenchymal stem cell.

**Figure 2:**
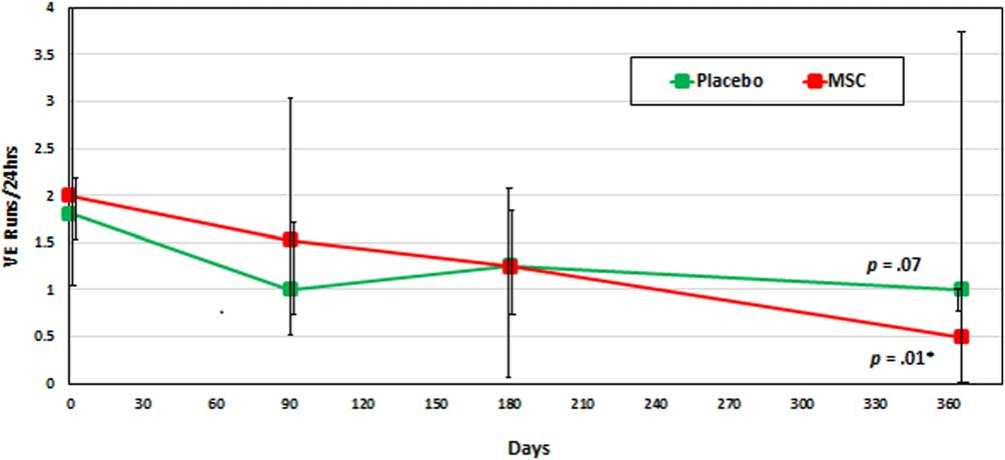
Change in high‐burden ventricular ectopy runs (≥1 VE Run/24 hours) over 12 months. In a subgroup analysis of patients with ≥ 1 VE Run in a 24‐hour period, there was a significant decrease from baseline to 12 months in the MSC group, but not in the placebo group (*p* = .01). The BMC group was excluded due to an insufficient number of runs for comparison. Of note, an intergroup comparison between the placebo and MSC groups did not reach statistical significance with *p* = .18. The baseline to 12‐month analysis within each group was performed using Wilcoxon Signed‐Rank tests, and the Kruskal–Wallis test was used for intergroup comparison. Abbreviation: MSC, mesenchymal stem cell.

At baseline, the BMC group had lower median log VE/24 hours compared with the other groups (Table 2; *p* = .01). Nevertheless, there was no significant short‐term increase or decrease in ventricular ectopy in any group (Fig. 1) within the first 3 days of implantation. There was also no significant change in ventricular ectopy between baseline and 1‐year follow‐up in any group. In the MSC group, log VE/24 hours at baseline was a median of 3.0 [2.3–3.4] and at one year was a median of 2.5 [2.0–3.1; *p* = .25]. In the subgroup analysis of patients with ≥1 VE runs demonstrated a decrease from 2.0 [1.0–10.8] runs to 0.5 [0.0–3.8] runs in the MSC group (*p*=0.01) and no significant change in the placebo group (1.8 [1.5–2.1] to 1.0 [0.8–1.0] *p* = .07; intergroup comparison: *p* = .18) (Fig. 2). An insufficient number of patients with ≥1 VE runs in the BMC group precluded analysis.

**Table 2:** Baseline ventricular ectopy

	Placebo	BMC	Total MSC	*p* value
Log (VE/24 hours)	3.2	2.2	3.0	.01^a^
(Median, [IQR])	[2.5–3.3]	[1.5–2.6]	[2.3–3.4]	
				
Ventricular ectopy runs	0.0	0.0	0.5	.02^b^
(Median, [IQR])	[0.0–1.0]	[0.0–0.0]	[0.0–1.5]	
# Complexes in longest ventricular ectopy run (Median, [IQR])	4.0 [3.0–5.8]	3.0 [3.0–3.5]	4.5 [3.0–7.0]	.29

a An intergroup comparison of median ventricular ectopy in a 24‐hour period amongst the 3 groups is significant (*p* = .01); BMC versus placebo (*p* = .005), BMC versus MSC (*p* = .007), MSC versus placebo (*p* = .49)

b An intergroup comparison of median ventricular ectopy runs between the 3 groups is significant at baseline (*p* = .02), but *p* only remains significant between the BMC and MSC groups (*p* = .007).

Abbreviation: VE, ventricular ectopy.

**Figure 3:**
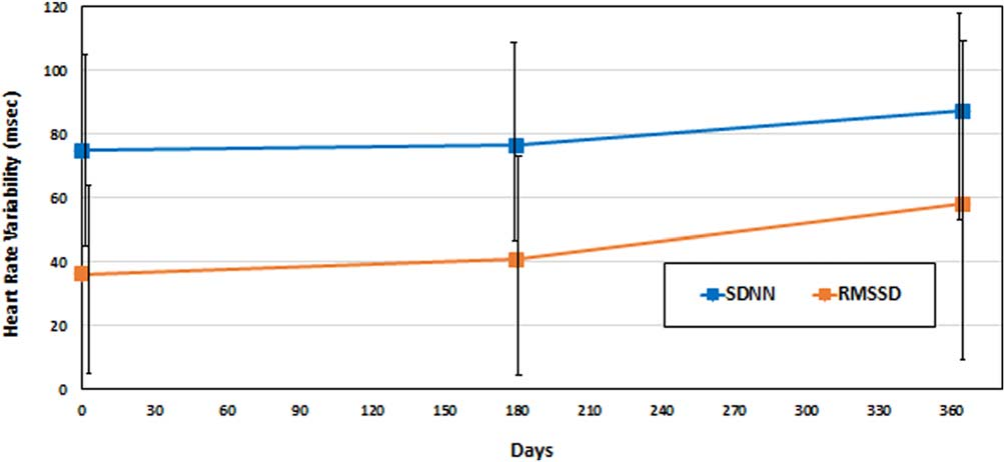
Change in heart rate variability (HRV) from baseline to 12‐month follow up. MSC patients (*N* = 21) from the POSEIDON trial were involved in HRV analysis. Changes in SDNN and RMSSD are shown. SDNN improved by 2% at 6 months (*p* = .19) and 16% at 12 months (*p* = .02). RMSSD improved by 13% at 6 months (*p* = .10) and 60% at 12 months (*p* = .01). Abbreviations: RMSSD, root mean square of the difference between coupling intervals of adjacent R‐R intervals; SDNN, standard deviation of all normal R‐R intervals.

In the HRV analysis with a total of 21 patients, mean baseline heart rate was 76 ± 8.2/minute and did not change at 12 months (74 ± 8.2/minute; *p* = .30). However, mean SDNN was 75 ± 30 msec and increased by 16% to 87 ± 32 msec after 12 months (*p* = .024). Mean RMSSD was 36.3 ± 30 msec at baseline and increased by 60% to 58.2 ± 50 msec at 12 months (*p* = .014) (Fig. 3). The increases in SDNN and RMSSD were observed in the subgroup of patients with any increase in EF (Table 3). Furthermore, improvement in RMSSD was also significantly associated in subgroups of patients with decrease in end diastolic volume and sphericity index (Table 3).

**Table 3:** Change in heart rate variability relative to change in ventricular function and volume (baseline to 12 months)

		SDNN		RMSSD	
	*N*	Increase	*p* value	Increase	*p* value
Entire cohort	21	21%	.02	35%	.01
Increase in EF	14	22%	.02	80%	.01
No improvement in EF	7	7%	.56	30%	.45
Decrease in EDV by ≥ 5%	10	22%	.11	100%	.03
Decrease in EDV by < 5%	11	12%	.11	32%	.24
Decrease in ESV by ≥ 5%	9	15%	.16	45%	.20
Decrease in ESV by < 5%	12	18%	.10	84%	.02
Decrease in SI by ≥ 10%	12	21%	.13	90%	.02
Decrease in SI by < 10%	9	3%	.09	31%	.32

Heart rate variability was performed on 21 patients in the POSEIDON study. *N* represents the number of patients in each cohort, and the percentages represent the increases in SDNN and RMSSD, respectively, from baseline to 12 months.

Abbreviations: EF, ejection fraction; EDV, end‐diastolic volume; ESV, end‐systolic volume; SI, sphericity index; SDNN, standard deviation of all normal sinus R‐R intervals; RMSSD, root mean square of the difference between coupling intervals of adjacent R‐R intervals.

**Figure 4:**
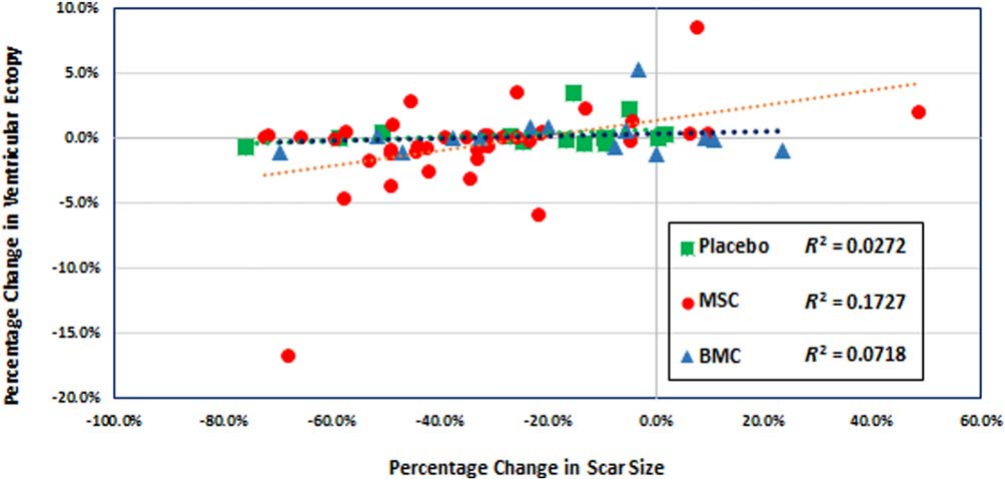
Correlation between percentage change in scar size and percentage change in ventricular ectopy in MSC, BMC, and placebo groups. There is a significant correlation of percentage change in scar size to percentage change in ventricular ectopy in the MSC group (*R* ^2^ = .17, *p* = .01) whereas there was no correlation found in the BMC group (*R* ^2^ = .07, *p* = .38) or in the Placebo group (*R* ^2^ = .03, *p* = .56). Abbreviations: BMC, bone marrow mononuclear cells; MSC, mesenchymal stem cell.

Scar mass decreased in the MSC group by 33.1% (95% CI, −40.7% to −25.5%) compared with 23.1% (95% CI, −36.4% to −9.7%) in the BMC group and 15.3% (95% CI, −29.3% to −1.3%) in the placebo group at 12 months (intergroup comparison *p* = .03; MSC vs. Placebo *p* = .02; MSC vs. BMC *p* = .08). There was a significant correlation of percentage change in scar size to percentage change in ventricular ectopy in the MSC group (*R* ^2^ = .17, *p* = .01) whereas there was no correlation found in the BMC group (*R* ^2^ = .07, *p* = .38) or in the Placebo group (*R* ^2^ = .03, *p* = .56) (Fig. 4).

## Discussion

The main finding from this study is that TESI with MSCs was not associated with short‐term or long‐term ventricular proarrhythmia among ICM patients. Moreover, we identified signals for potential antiarrhythmic effect or at least improvement in risk factors for ventricular arrhythmias in the patients who had MSC injection. In the BMC and placebo groups, no similar signal for improvement was noted.

Cell‐based therapies for cardiac repair have progressed from proof of concept to randomized clinical trials demonstrating significant left ventricular improvement. The recently published ixCELL‐DCM study showed reduction of clinical cardiac events in a randomized controlled study evaluating TESI of two types of BMCs 22. Nevertheless, the field of cardiac stem cell therapy continues to engender controversy, particularly via the concern of potential arrhythmogenic effects 23. Early preclinical work demonstrated both pro‐ and anti‐arrhythmic effects 15, 16, 17, 24, 25. The types of cardiac stem cell (i.e., skeletal myoblasts, bone marrow‐derived cells, etc.) used in these preclinical studies, as well as the different routes of administration (intracoronary, intraventricular), may account for varied results.

In early human clinical studies with injection of stem cells into the heart, there was concern for ventricular arrhythmia. Menashe et al. showed that four of nine surviving patients with skeletal myoblast implants at the time of bypass surgery developed VT 26. Another study also revealed potential ventricular proarrhythmic effects from skeletal myoblast transplantation at the time of bypass surgery 27. These early studies set a precedent that stem cell implants may be proarrhythmic. Furthermore, there were plausible mechanisms to explain proarrhythmia. Stem cell implants could set up areas of heterogeneous conduction and repolarization, which could serve as a substrate for ventricular dysrhythmias.

Recent human clinical studies using either BMCs or MSCs have shown neither pro‐ nor antiarrhythmic effects from stem cell implantation. In fact, there have been no clinical studies of stem cells implanted in the heart designed to prospectively test antiarrhythmic effects. In one study, autologous mononuclear bone marrow cells did not demonstrate any ventricular proarrhythmia, although indices of parasympathetic tone were suppressed 28. In the BOOST randomized clinical study, there was no difference in ambulatory monitor ventricular ectopy burden, nonsustained VT, or inducible VT in patients treated after acute MI with autologous BMCs as compared with control patients 29. Beeres et al. demonstrated that intramyocardial BMC injection does not induce ventricular arrhythmias nor does it alter electrophysiological properties 18. Furthermore, Huikuri et al. established neutral arrhythmogenic effects from intracoronary injection of BMCs in patients treated post STEMI 19. Conversely, Hare et al. noted a reduction in monitored NSVT episodes in an early study of intravenous allogeneic hMSCs 30. Finally, the recently published MSC‐HF trial showed similar rates of VT/VF (5%) in MSC injected patients and placebo injected patients 31. Though BMCs have been shown to be safe for patients with chronic ICM in the TAC‐HFT trial, they were not shown to be more efficacious than placebo in terms of decreasing infarct size and improving regional myocardial function. For this reason, we decided to focus on MSCs for this post hoc analysis despite certain advantages only afforded with BMCs.

By combining the data sets from the POSEIDON and TAC‐HFT studies, we studied potential arrhythmogenic effects of autologous and allogeneic MSCs in one of the largest groups of patients to date. No proarrhythmic effects from MSC implant occurred either in the short‐term (within days) as a result of the injury or long‐term (up to 1 year). Furthermore, there was no evidence that MSC implant increased ventricular arrhythmias or worsened markers of ventricular arrhythmias. Although the changes observed in our post hoc analysis for ventricular ectopy in the MSC group were not statistically significant, there was a consistent trend toward improvement. This may relate to the small sample size and the overall low burden of ventricular arrhythmias. Further studies are needed to better address this question.

Moreover, MSC implantation was associated with scar size reduction 6. In addition, scar size has been correlated with ventricular arrhythmias in ICM 30. In our study, we identified a significant but weak correlation in the patients who received MSCs between percentage change in scar size from baseline to 12 months and percentage change in ventricular ectopy over the same time period. This correlation was not present for the BMC or Placebo groups. As scar and peri‐infarct ischemic tissue plays a large role in VT in patients with ICM, any potential antiarrhythmic effects from MSC may be mitigated in part by scar reduction. Other possible mechanisms for reduction in ventricular arrhythmia may be due to reduction in areas of heterogeneous conduction through direct injection of stem cells in peri‐infarct areas, or conversely, improved cell‐cell coupling and positive remodeling may lead to enhanced myocardial function. In addition, the secretion of paracrine factors from MSCs should not be overlooked as a potential mechanism. These protective paracrine effects have been shown to have important roles in cardiac repair and regeneration which can in turn influence cardiac remodeling. In these trials, relevant paracrine growth factors were not measured, but this may be important to consider in future studies.

The limitations of this study are important to consider, as this was a post hoc analysis of two separate, randomized clinical trials. Therefore, the patient populations and therapies differed. Patients in both trials were medically optimized with evidence‐based therapies (statins, aspirin, beta blockers, and ACE inhibitors/ARBs), counseled regularly about lifestyle choices, and followed closely during the 1‐year period, and these therapies alone could have contributed to clinical improvement. Also, both trials were Phase I/II studies to examine the safety and efficacy of different types (allogeneic/autologous) and doses of MSC, and only one study had a placebo control and a BMC group. Furthermore, the patients in these studies had relatively low VE burden and no recent VT episodes. Therefore, it would be difficult to prove an antiarrhythmic effect from MSC implant in a group that was not having significant or sufficient ventricular ectopy or tachyarrhythmias.

## Conclusion

In conclusion, transendocardial injection of MSCs to the border zone of infarcted myocardium in patients with ICM was not associated with short‐term (days) or long‐term (one year) ventricular proarrhythmia. In a group of patients selected with low ventricular ectopy burden, there were signals of improvement in either ventricular arrhythmias or markers of ventricular arrhythmias. Prospective studies may clarify the role of TESI with MSCs to reduce ventricular arrhythmias.

## Author Contributions

A.R. and C.R.B.: conception and design, collection and/or assembly of data, data analysis and interpretation, manuscript writing; A.M.M. and J.J.G.: data analysis and interpretation, final approval of manuscript; D.L.D.: administrative support, provision of study material or patients, final approval of manuscript; C.H., V.G., and Y.A.: collection and/or assembly of data, final approval of manuscript; J.O.C. and J.F.V.‐G.: final approval of manuscript; A.W.H.: provision of study material or patients, final approval of manuscript; R.J.M.: conception and design, final approval of manuscript; J.M.H.: financial support, administrative support, provision of study material or patients, final approval of manuscript; R.D.M.: conception and design, administrative support, collection and/or assembly of data, data analysis and interpretation, manuscript writing, final approval of manuscript.

## Disclosure of Potential Conflicts of Interest

A.M.M.: employed by Emmes Corporation; D.L.D: consulting fees from Biocardia, Inc., BDS, and Longeveron LLC; A.W.H.—Ownership interest in Vestion, Inc.; J.M.H.—Ownership interest in Vestion, Inc. The other authors indicated no potential conflicts of interest.
